# A national survey assessing public readiness for digital health strategies against COVID-19 within the United Kingdom

**DOI:** 10.1038/s41598-021-85514-w

**Published:** 2021-03-16

**Authors:** Viknesh Sounderajah, Jonathan Clarke, Seema Yalamanchili, Amish Acharya, Sheraz R. Markar, Hutan Ashrafian, Ara Darzi

**Affiliations:** 1 Department of Surgery and Cancer, Imperial College London, London, W2 1NY UK; 2 Institute of Global Health Innovation, Imperial College London, 10th Floor, Queen Elizabeth Queen Mother building, St Mary’s Hospital Campus, Praed Street, London, W2 1NY UK; 3 Department of Mathematics, Imperial College London, London, SW7 2AZ UK

**Keywords:** Health policy, Public health

## Abstract

There is concern that digital public health initiatives used in the management of COVID-19 may marginalise certain population groups. There is an overlap between the demographics of groups at risk of digital exclusion (older, lower social grade, low educational attainment and ethnic minorities) and those who are vulnerable to poorer health outcomes from SARS-CoV-2. In this national survey study (n = 2040), we assessed how the UK population; particularly these overlapping groups, reported their preparedness for digital health strategies. We report, with respect to using digital information to make health decisions, that those over 60 are less comfortable (net comfort: 57%) than those between 18 and 39 (net comfort: 78%) and lower social grades are less comfortable (net comfort: 63%) than higher social grades (net comfort: 75%). With respect to a preference for digital over non-digital sources in seeking COVID-19 health information, those over 60 (net preference: 21%) are less inclined than those between 18 and 39 (net preference: 60%) and those of low educational attainment (net preference: 30%) are less inclined than those of high educational attainment (net preference: 52%). Lastly, with respect to distinguishing reliable digital COVID-19 information, lower social grades (net confidence: 55%) are less confident than higher social grades (net confidence: 68%) and those of low educational attainment (net confidence: 51%) are less confident than those of high educational attainment (net confidence: 71%). All reported differences are statistically significant (*p* < 0.01) following multivariate regression modelling. This study suggests that digital public health approaches to COVID-19 have the potential to marginalise groups who are concurrently at risk of digital exclusion and poor health outcomes from SARS-CoV-2.

## Introduction

As of 23nd December 2020, the SARS-CoV-2 virus has infected over 75.9 million people and has claimed over 1.74 million lives globally^1^. Throughout, the World Health Organization has emphasised the importance of strict and prompt compliance with public health strategies as the cornerstone in addressing the COVID-19 pandemic^2^. As such, governments have mandated nationwide and regional measures, including social distancing, quarantining, testing and contact tracing^3^. However, for these approaches to be effective, all sections of the population need to be included in communication efforts.

UK health bodies have been moving towards a ‘digital first’ strategy as a means of improving healthcare accessibility. This has led to the integration of digital technologies into various elements of national and regional public health plans. These have been especially focussed around the dissemination of critical health information, disease surveillance and digital contact tracing^4^.

Whilst digital technologies can improve the speed, reach and cost efficiency of many traditional public health measures, there are also well described barriers to their use, which can lead to the digital exclusion of population subsets. These barriers^5–7^ can be broadly categorised as:

1. Access—availability and affordability of internet connection and/or equipment, such as laptops or personal computers, smartphones, tablets or smartwatches.
2. Skills—deficits in knowledge or ability to use digital resources.
3. Engagement—further factors impeding digital interaction, even in the presence of adequate access and skills (e.g., confidence, motivation or time opportunity).

According to the UK Office for National Statistics (ONS), access has steadily increased, with 96% of households with internet connectivity in 2020. Conversely, the same data suggests there remain significant disparities with respect to the skills to make use of this access^8–10^. The need for reduced in-person contact during the COVID-19 pandemic has fast-tracked the integration and use of digital services by some sectors of the public. Those who have found themselves unable to utilise such services are at highest risk of digital exclusion. These sections of the population include those who are older, are of a lower social grade, have lower educational attainment, have disabilities and those who do not use English as a first language^11^.

Worryingly, mortality and excess deaths from COVID-19 have been higher in the UK compared to other European countries^12^. Greater susceptibility to COVID-19 in the UK has been associated with increased age, socioeconomic deprivation, comorbidity and ethnicity; predominantly those of Afro-Caribbean and South Asian origin^13^. Strikingly, there is significant overlap between these medically vulnerable groups and the aforementioned populations at the highest risk of digital exclusion. This combination of the direct health impact of COVID-19, and the transition towards a digital-first management strategy, therefore, poses a threat of deepening the digital divide thus impeding access, engagement and the efficacy of health services^14,15^**.** Accordingly, the failure to account for groups at risk of digital exclusion will likely compound health and societal inequalities.

To date, research has not investigated whether members of the UK population—particularly members who identify with at-risk socio-demographic groups—are in a position to participate in digital health strategies. Do members of the population possess adequate access to digital devices and harbour sufficient confidence in digitally transmitted information for digital health strategies to be effective? Moreover, which sources of information do members of the population access, to what degree are those sources trusted, and how does the population view the particularly important information source of contact-tracing applications? To answer these questions, we conducted a national survey that asked individuals to report their access to digital devices and their perceptions about digital information relevant to the UK’s digital health strategies.

## Methods

### Survey development

An online survey was co-designed with qualitative experts from YouGov (YouGov PLC, London, UK), a market research company. Existing frameworks were identified through a literature search to provide the foundation to the survey design. The eHealth Literacy Framework^16^ was the only relevant validated framework identified which covers access, education and engagement as barriers to digital inclusion. It consists of seven core domains.

Thereafter, the UK public health response to COVID-19 was assessed for features and strategies utilising a digital approach. These included delivery of information around the virus, public health messaging about social distancing and quarantine precautions, symptom tracking and contact tracing. These features were mapped to the eHealth Literacy Framework to devise a set of 17 core questions. (Appendix 1).

These were grouped into five themes in keeping with the study objectives: (1) access to personal digital devices (2) confidence to independently source and use information from digital technologies to answer health related questions, (3) identifying which sources of information are commonly used in gathering COVID-19 specific health information, (4) identifying which sources of information harbour the most trust in gathering COVID-19 specific health information and (5) quantifying public opinion regarding the use of the contact tracing apps.

### Sample

A sample of 2040 adults was achieved through YouGov’s non-probabilistic sampling method. YouGov employ an active sampling methodology to ensure that there is adequate socio-demographic representation within their respondents^17^. The proportions of demographics within the respondent panel are compared against (1) UK census data from 2011, (2) large scale random probability surveys (e.g., Labour Force Survey, The National Readership survey and the British Election Study), (3) results of the 2017 general election and 2016 referendum and (4) ONS population estimates^18^. This ensures that the coverage is representative of the population as a whole as opposed to those with internet or telephone access. The attained sample is retrieved from a larger panel of more than 360,000 adults, who are registered and incentivised to participate in surveys^18^. The sample is representative of UK adults in terms of gender, age, ethnicity, social grade, education attainment and geographical region of residence.

Data was collected between the dates of 15th June 2020 and 24th June 2020 via an online survey conducted by YouGov. A sample size calculation was not performed due to the absence of appropriate pilot data upon which a reliable power calculation may be based. Participants were identified from the YouGov panel and were sent an e-mail with a survey link. Whilst this mode of dissemination does introduce bias, there are numerous reports to suggest that the views of those with access to the internet are similar from those without^19^. Moreover, it has been noted that response rates for telephone polls have been sharply declining in recent years; strikingly below 10% in inner city regions^18^.

YouGov do not provide response rates for individual datasets, however, it is noted that their aggregate response rate is typically between 35 and 50%; a figure that varies based upon subject matter, complexity and length of survey. All invited participants are from a panel of over 800,000 adults who have registered to participate in surveys and the responding sample is weighted to the profile of the sample definition in order to provide a representative reporting sample. Of note, a Pew Research Center Report^20^ states that YouGov ‘consistently outperformed’ other vendors of nonprobability surveys with regards to accuracy of population representation. As such, given the study goal of rapidly attaining data during a pandemic period, it was felt that an online dissemination strategy, coupled with careful socio-demographic sampling, would allow for accurate yet pragmatic data collection.

### Data analysis

We utilised descriptive statistics to describe the sample by gender, age, ethnicity, social grade, educational attainment and governmental office region respectively. Social grade was categorised using the National Readership Survey (NRS) classification system and dichotomised into ‘middle class’ (ABC1) and ‘working class’ (C2DE) groups^21^. Education was classified as ‘low’ (GCSE attainment or below), ‘medium’ (A-level or equivalent attainment) and ‘high’ (university degree attainment and above). Respondent ages were grouped into young adults (18–39 years), middle-aged (40–59 years) and elderly (60+ years). Ethnicity is classed as either Caucasian or Black, Asian and minority ethnic (BAME). Government Office regions were aggregated to Southern England (London, South East and South West), Midlands (East of England, East Midlands and West Midlands), Northern England (Yorkshire and the Humber, North East and North West) and Devolved Nations (Scotland, Wales and Northern Ireland).

### Outcome

For questions with Likert-type ordinal responses, ordinal logistic regression was performed to examine the relationships between responses and the panel of demographic characteristics described above. Binary logistic regression was used for questions with binary responses. Brant tests were performed to assess the proportional odds assumption for each ordinal logistic regression model using the Stata *omodel* and *brant* commands.

In order to identify discrete response types within survey domains, K-means clustering was applied to all Likert-type ordinal response variables in each domain. Data were normalised by min–max transformation and optimal clusters sizes were determined by relative maxima in silhouette and Calinski Harabasz scores and relative minima in Davies–Bouldin scores^22–24^. The responses of each cluster and their demographic characteristics were described. All analyses were undertaken on Stata/SE 16.0 (Stata Corporation LP, College Station, Texas, United States of America). K-means clustering was performed using Python v.3.6.8 with the scikit-learn library (version 0.23.1).

### Ethical approval

This study was waived by our University Research Office (Ruth Nicholson (Head of Research Governance and Integrity)), in accordance with UK HRA guidelines, as this study is a non-clinical population survey audit of public respondents (involving neither identifiable information, patients nor vulnerable individuals) that constitutes an observation of usual practice. Informed consent was attained from all participants of the survey by YouGov as part of their survey process. YouGov provided the datasets to The Institute of Global Health Innovation and the data is publicly available upon request. Patients and members of the public were not involved in the design, reporting or conduct of the study.

## Results

**Figure 1:**
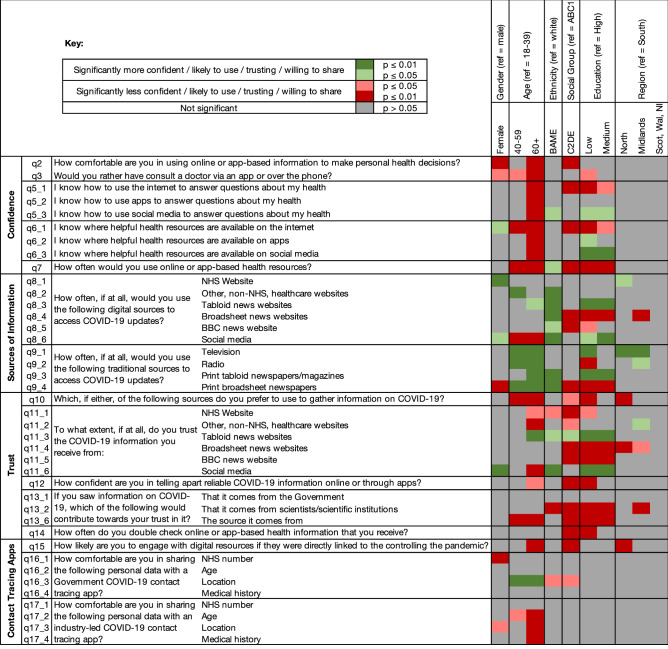
A significance map detailing directionality and significance of relationships between responses and the panel of demographic characteristics.

A sample of 2040 adults (Table 1) was achieved. Figure 1 is a significance map which details the directionality and the level of significance associated with responses and the panel of pre-specified demographic characteristics. The results from the logistic regression analyses are detailed in Table 2.

**Table 1:** Survey respondent demographics table.

Total	Number (n = 2040)	Percentage within YouGov sample (%)
**Gender**		
Male	990	49
Female	1050	51
**Age**		
18–29	377	18
30–39	369	18
40–49	347	17
50–59	284	15
60–69	356	18
70+	259	14
**Social grade**		
AB	571	28
C1	592	29
C2	428	21
DE	449	22
**Educational attainment**		
Low	535	26
Medium	871	43
High	634	31
**Region**		
North East	72	4
North West	225	11
Yorkshire and the Humber	178	9
East Midlands	162	8
West Midlands	164	8
East of England	168	8
London	268	13
South East	285	14
South West	191	9
Wales	98	5
Scotland	172	8
Northern Ireland	57	3
**Ethnicity**		
White	1754	86
BAME	286	14

**Table 2:** Tables demonstrating the results of the multivariate regression analyses for survey questions.

q2	How comfortable are you in using online or app-based information to make personal health decisions?						
		Coeff	SE	Z score	*p* value	95% Confidence Interval	
Gender	Male	Reference					
	Female	0.2380	0.0850	2.80	0.005	0.0714	0.4046
Age group	18–39	Reference					
	40–59	0.1716	0.1075	1.60	0.111	− 0.0392	0.3824
	60 +	0.9757	0.1121	8.70	0.000	0.7560	1.1954
Ethnicity	White	Reference					
	BAME	0.0670	0.1268	0.53	0.597	− 0.1814	0.3155
Social group	ABC1	Reference					
	C2DE	0.5096	0.0933	5.46	0.000	0.3268	0.6924
Education	Low	0.1148	0.1240	0.93	0.355	− 0.1283	0.3579
	Medium	0.0866	0.0978	0.89	0.376	− 0.1051	0.2782
	High	Reference					
Region	South	Reference					
	North	0.0370	0.1113	0.33	0.739	− 0.1811	0.2552
	Midlands	0.0612	0.1085	0.56	0.573	− 0.1514	0.2738
	Scot, Wal, NI	− 0.0494	0.1307	− 0.38	0.706	− 0.3055	0.2067

q3	Would you rather have consult a doctor via an app or over the phone?						
		Coeff	SE	Z score	*p* value	95% Confidence interval	
Gender	Male	Reference					
	Female	0.2540	0.1087	2.34	0.019	0.0410	0.4671
Age group	18–39	Reference					
	40–59	0.2679	0.1265	2.12	0.034	0.0199	0.5159
	60 +	1.2091	0.1520	7.96	0.000	0.9113	1.5070
Ethnicity	White	Reference					
	BAME	− 0.0726	0.1490	− 0.49	0.626	− 0.3647	0.2194
Social group	ABC1	Reference					
	C2DE	0.1437	0.1218	1.18	0.238	-0.0951	0.3825
Education	Low	0.4189	0.1689	2.48	0.013	0.0879	0.7498
	Medium	0.0857	0.1208	0.71	0.478	-0.1511	0.3226
	High	Reference					
Region	South	Reference					
	North	0.0759	0.1442	0.53	0.599	− 0.2067	0.3585
	Midlands	− 0.0185	0.1385	− 0.13	0.894	− 0.2901	0.2530
	Scot, Wal, NI	0.0551	0.1714	0.32	0.748	− 0.2809	0.3910

q5_1	I know how to use the internet to answer questions about my health						
		Coeff	SE	Z score	*p* value	95% Confidence interval	
Gender	Male	Reference					
	Female	− 0.1000	0.0867	− 1.15	0.249	− 0.2700	0.0700
Age Group	18–39	Reference					
	40–59	0.0396	0.1096	0.36	0.718	− 0.1752	0.2544
	60 +	0.3839	0.1132	3.39	0.001	0.1621	0.6057
Ethnicity	White	Reference					
	BAME	0.0752	0.1307	0.58	0.565	− 0.1809	0.3313
Social Group	ABC1	Reference					
	C2DE	0.2718	0.0954	2.85	0.004	0.0848	0.4589
Education	Low	0.4670	0.1281	3.65	0.000	0.2160	0.7180
	Medium	0.2097	0.0995	2.11	0.035	0.0147	0.4047
	High	Reference					
Region	South	Reference					
	North	0.1365	0.1131	1.21	0.228	− 0.0852	0.3582
	Midlands	− 0.0413	0.1108	− 0.37	0.709	− 0.2584	0.1757
	Scot, Wal, NI	− 0.0484	0.1355	− 0.36	0.721	− 0.3141	0.2172

q5_2	I know how to use apps to answer questions about my health						
		Coeff	SE	Z score	*p* value	95% Confidence interval	
Gender	Male	Reference					
	Female	0.0413	0.0825	0.50	0.616	− 0.1203	0.2030
Age group	18–39	Reference					
	40–59	0.0393	0.1041	0.38	0.706	− 0.1648	0.2434
	60 +	0.7316	0.1089	6.72	0.000	0.5181	0.9450
Ethnicity	White	Reference					
	BAME	− 0.2210	0.1227	− 1.80	0.072	− 0.4616	0.0195
Social group	ABC1	Reference					
	C2DE	0.1257	0.0908	1.38	0.166	− 0.0523	0.3037
Education	Low	− 0.1335	0.1218	− 1.10	0.273	− 0.3723	0.1052
	Medium	− 0.1332	0.0951	− 1.40	0.161	− 0.3197	0.0532
	High	Reference					
Region	South	Reference					
	North	0.0389	0.1079	0.36	0.719	− 0.1726	0.2504
	Midlands	− 0.0303	0.1053	− 0.29	0.773	− 0.2367	0.1760
	Scot, Wal, NI	0.0197	0.1301	0.15	0.880	− 0.2354	0.2748

q5_3	I know how to use social media to answer questions about my health						
		Coeff	SE	Z score	*p* value	95% Confidence interval	
Gender	Male	Reference					
	Female	− 0.0831	0.0819	− 1.01	0.311	− 0.2436	0.0775
Age Group	18–39	Reference					
	40–59	− 0.1349	0.1030	− 1.31	0.190	− 0.3367	0.0669
	60 +	0.5348	0.1075	4.98	0.000	0.3241	0.7455
Ethnicity	White	Reference					
	BAME	− 0.2885	0.1219	− 2.37	0.018	− 0.5275	− 0.0495
Social Group	ABC1	Reference					
	C2DE	0.0310	0.0900	0.34	0.731	− 0.1454	0.2074
Education	Low	− 0.2758	0.1212	− 2.28	0.023	− 0.5134	− 0.0382
	Medium	− 0.2245	0.0942	− 2.38	0.017	− 0.4090	− 0.0399
	High	Reference					
Region	South	Reference					
	North	− 0.0997	0.1072	− 0.93	0.352	− 0.3098	0.1103
	Midlands	− 0.1976	0.1049	− 1.88	0.060	− 0.4032	0.0080
	Scot, Wal, NI	− 0.2346	0.1282	− 1.83	0.067	− 0.4860	0.0167

q6_1	I know where helpful health resources are available on the internet						
		Coeff	SE	Z score	*p* value	95% Confidence interval	
Gender	Male	Reference					
	Female	− 0.2090	0.0860	− 2.43	0.015	− 0.3776	− 0.0403
Age group	18–39	Reference					
	40–59	0.3339	0.1091	3.06	0.002	0.1201	0.5477
	60 +	0.7773	0.1132	6.87	0.000	0.5554	0.9992
Ethnicity	White	Reference					
	BAME	0.0746	0.1282	0.58	0.560	− 0.1765	0.3258
Social group	ABC1	Reference					
	C2DE	0.2989	0.0950	3.15	0.002	0.1128	0.4850
Education	Low	0.5056	0.1270	3.98	0.000	0.2566	0.7545
	Medium	0.2461	0.0988	2.49	0.013	0.0525	0.4397
	High	Reference					
Region	South	Reference					
	North	0.1140	0.1125	1.01	0.311	− 0.1065	0.3345
	Midlands	0.1105	0.1096	1.01	0.313	− 0.1043	0.3254
	Scot, Wal, NI	− 0.0039	0.1347	− 0.03	0.977	− 0.2679	0.2601

q6_2	I know where helpful health resources are available on apps						
		Coeff	SE	Z score	*p* value	95% Confidence interval	
Gender	Male	Reference					
	Female	0.0335	0.0818	0.41	0.682	− 0.1269	0.1939
Age group	18–39	Reference					
	40–59	0.1526	0.1040	1.47	0.142	− 0.0511	0.3564
	60 +	0.8143	0.1089	7.47	0.000	0.6007	1.0278
Ethnicity	White	Reference					
	BAME	− 0.2085	0.1220	− 1.71	0.087	− 0.4477	0.0306
Social group	ABC1	Reference					
	C2DE	0.0864	0.0906	0.95	0.340	− 0.0911	0.2639
Education	Low	− 0.2939	0.1210	− 2.43	0.015	− 0.5310	− 0.0567
	Medium	− 0.1733	0.0950	− 1.82	0.068	− 0.3595	0.0129
	High	Reference					
Region	South	Reference					
	North	0.0173	0.1071	0.16	0.872	− 0.1926	0.2273
	Midlands	− 0.0213	0.1050	− 0.20	0.839	− 0.2271	0.1845
	Scot, Wal, NI	0.0459	0.1278	0.36	0.720	− 0.2046	0.2964

q6_3	I know where helpful health resources are available on social media						
		Coeff	SE	Z score	*p* value	95% Confidence interval	
Gender	Male	Reference					
	Female	− 0.1278	0.0819	− 1.56	0.119	− 0.2883	0.0327
Age group	18–39	Reference					
	40–59	− 0.0388	0.1035	− 0.37	0.708	− 0.2416	0.1640
	60 +	0.6177	0.1078	5.73	0.000	0.4064	0.8289
Ethnicity	White	Reference					
	BAME	− 0.2338	0.1205	− 1.94	0.052	− 0.4700	0.0023
Social group	ABC1	Reference					
	C2DE	− 0.0543	0.0904	− 0.60	0.548	− 0.2315	0.1229
Education	Low	− 0.4383	0.1210	− 3.62	0.000	− 0.6755	− 0.2011
	Medium	− 0.3138	0.0947	− 3.31	0.001	− 0.4993	− 0.1282
	High	Reference					
Region	South	Reference					
	North	− 0.1324	0.1072	− 1.24	0.217	− 0.3425	0.0777
	Midlands	− 0.1459	0.1051	− 1.39	0.165	− 0.3519	0.0600
	Scot, Wal, NI	− 0.1331	0.1271	− 1.05	0.295	− 0.3822	0.1159

q7	How often would you use online or app-based health resources?						
		Coeff	SE	Z score	*p* value	95% Confidence interval	
Gender	Male	Reference					
	Female	− 0.1314	0.0843	− 1.56	0.119	− 0.2966	0.0339
Age group	18–39	Reference					
	40–59	0.3121	0.1050	2.97	0.003	0.1064	0.5178
	60 +	1.0752	0.1111	9.67	0.000	0.8573	1.2930
Ethnicity	White	Reference					
	BAME	− 0.2583	0.1249	− 2.07	0.039	− 0.5032	− 0.0134
Social group	ABC1	Reference					
	C2DE	0.4089	0.0932	4.39	0.000	0.2262	0.5915
Education	Low	0.5550	0.1251	4.43	0.000	0.3097	0.8002
	Medium	0.2614	0.0959	2.72	0.006	0.0733	0.4494
	High	Reference					
Region	South	Reference					
	North	0.0325	0.1112	0.29	0.770	− 0.1855	0.2505
	Midlands	0.1058	0.1080	0.98	0.327	− 0.1059	0.3176
	Scot, Wal, NI	− 0.0170	0.1304	− 0.13	0.897	− 0.2725	0.2386

q8_1	How often, if at all, would you use the following digital sources to access COVID-19 updates?					NHS website	
		Coeff	SE	Z score	*p* value	95% Confidence interval	
Gender	Male	Reference					
	Female	− 0.4827	0.1039	− 4.65	0.000	− 0.6864	− 0.2791
Age group	18–39	Reference					
	40–59	0.1219	0.1203	1.01	0.311	− 0.1138	0.3576
	60 +	0.1575	0.1354	1.16	0.245	− 0.1079	0.4230
Ethnicity	White	Reference					
	BAME	0.0280	0.1414	0.20	0.843	− 0.2492	0.3051
Social group	ABC1	Reference					
	C2DE	0.1640	0.1169	1.40	0.161	− 0.0651	0.3932
Education	Low	0.3073	0.1602	1.92	0.055	− 0.0067	0.6213
	Medium	0.1465	0.1128	1.30	0.194	− 0.0745	0.3675
	High	Reference					
Region	South	Reference					
	North	− 0.2649	0.1339	− 1.98	0.048	− 0.5274	− 0.0025
	Midlands	− 0.0843	0.1320	− 0.64	0.523	− 0.3429	0.1743
	Scot, Wal, NI	− 0.1974	0.1584	− 1.25	0.212	− 0.5078	0.1129

q8_2	How often, if at all, would you use the following digital sources to access COVID-19 updates?					Other, non-NHS, healthcare websites	
		Coeff	SE	Z score	*p* value	95% Confidence interval	
Gender	Male	Reference					
	Female	− 0.1185	0.1040	− 1.14	0.255	− 0.3224	0.0854
Age group	18–39	Reference					
	40–59	− 0.3477	0.1238	− 2.81	0.005	− 0.5903	− 0.1050
	60 +	0.0882	0.1368	0.65	0.519	− 0.1798	0.3563
Ethnicity	White	Reference					
	BAME	− 0.6298	0.1497	− 4.21	0.000	− 0.9232	− 0.3364
Social group	ABC1	Reference					
	C2DE	0.0081	0.1175	0.07	0.945	− 0.2222	0.2385
Education	Low	0.2563	0.1584	1.62	0.106	− 0.0542	0.5668
	Medium	− 0.0403	0.1158	− 0.35	0.727	− 0.2672	0.1865
	High	Reference					
Region	South	Reference					
	North	0.0566	0.1364	0.41	0.678	− 0.2108	0.3240
	Midlands	0.0759	0.1337	0.57	0.570	− 0.1861	0.3378
	Scot, Wal, NI	0.1476	0.1591	0.93	0.353	− 0.1642	0.4594

q8_3	How often, if at all, would you use the following digital sources to access COVID-19 updates?					Tabloid news websites	
		Coeff	SE	Z score	*p* value	95% Confidence interval	
Gender	Male	Reference					
	Female	0.0512	0.1082	0.47	0.636	− 0.1609	0.2633
Age group	18–39	Reference					
	40–59	− 0.1717	0.1300	− 1.32	0.187	− 0.4266	0.0832
	60 +	− 0.3291	0.1431	− 2.30	0.021	− 0.6094	− 0.0487
Ethnicity	White	Reference					
	BAME	− 0.6280	0.1490	− 4.21	0.000	− 0.9201	− 0.3359
Social group	ABC1	Reference					
	C2DE	− 0.1256	0.1212	− 1.04	0.300	− 0.3632	0.1121
Education	Low	− 0.6695	0.1652	− 4.05	0.000	− 0.9933	− 0.3458
	Medium	− 0.6271	0.1209	− 5.19	0.000	− 0.8641	− 0.3900
	High	Reference					
Region	South	Reference					
	North	− 0.0371	0.1417	− 0.26	0.794	− 0.3147	0.2406
	Midlands	0.0452	0.1397	0.32	0.747	− 0.2287	0.3190
	Scot, Wal, NI	0.3084	0.1678	1.84	0.066	− 0.0204	0.6372

q8_4	How often, if at all, would you use the following digital sources to access COVID-19 updates?					Broadsheet news websites	
		Coeff	SE	Z score	*p* value	95% Confidence interval	
Gender	Male	Reference					
	Female	0.1206	0.1028	1.17	0.241	− 0.0810	0.3222
Age group	18–39	Reference					
	40–59	− 0.0291	0.1214	− 0.24	0.810	− 0.2670	0.2087
	60 +	0.0158	0.1361	0.12	0.907	− 0.2510	0.2826
Ethnicity	White	Reference					
	BAME	− 0.4834	0.1453	− 3.33	0.001	− 0.7682	− 0.1986
Social group	ABC1	Reference					
	C2DE	0.3527	0.1173	3.01	0.003	0.1228	0.5827
Education	Low	1.0389	0.1606	6.47	0.000	0.7242	1.3537
	Medium	0.6026	0.1148	5.25	0.000	0.3776	0.8275
	High	Reference					
Region	South	Reference					
	North	0.2428	0.1353	1.80	0.073	− 0.0223	0.5080
	Midlands	0.4897	0.1329	3.68	0.000	0.2292	0.7502
	Scot, Wal, NI	0.2299	0.1578	1.46	0.145	− 0.0794	0.5393

q8_5	How often, if at all, would you use the following digital sources to access COVID-19 updates?					BBC news website	
		Coeff	SE	Z score	*p* value	95% Confidence interval	
Gender	Male	Reference					
	Female	0.0979	0.1023	0.96	0.339	− 0.1026	0.2984
Age group	18–39	Reference					
	40–59	0.0816	0.1198	0.68	0.496	− 0.1532	0.3164
	60 +	0.0223	0.1346	0.17	0.868	− 0.2414	0.2861
Ethnicity	White	Reference					
	BAME	− 0.3406	0.1427	− 2.39	0.017	− 0.6203	− 0.0609
Social group	ABC1	Reference					
	C2DE	0.4004	0.1177	3.40	0.001	0.1696	0.6312
Education	Low	0.3876	0.1578	2.46	0.014	0.0782	0.6970
	Medium	0.1006	0.1122	0.90	0.370	− 0.1193	0.3206
	High	Reference					
Region	South	Reference					
	North	0.0329	0.1335	0.25	0.805	− 0.2288	0.2946
	Midlands	− 0.0798	0.1312	− 0.61	0.543	− 0.3369	0.1772
	Scot, Wal, NI	0.0257	0.1567	0.16	0.870	− 0.2814	0.3328

q8_6	How often, if at all, would you use the following digital sources to access COVID-19 updates?					Social media	
		Coeff	SE	Z score	*p* value	95% Confidence interval	
Gender	Male	Reference					
	Female	− 0.2297	0.1032	− 2.23	0.026	− 0.4320	− 0.0274
Age group	18–39	Reference					
	40–59	0.3140	0.1209	2.60	0.009	0.0770	0.5510
	60 +	0.8784	0.1381	6.36	0.000	0.6078	1.1490
Ethnicity	White	Reference					
	BAME	− 0.5864	0.1430	− 4.10	0.000	− 0.8666	− 0.3062
Social group	ABC1	Reference					
	C2DE	− 0.1531	0.1162	− 1.32	0.187	− 0.3809	0.0746
Education	Low	− 0.3400	0.1588	− 2.14	0.032	− 0.6513	− 0.0287
	Medium	− 0.3084	0.1140	− 2.70	0.007	− 0.5319	− 0.0849
	High	Reference					
Region	South	Reference					
	North	0.0555	0.1357	0.41	0.683	− 0.2105	0.3214
	Midlands	0.0073	0.1327	0.05	0.956	− 0.2528	0.2674
	Scot, Wal, NI	− 0.0878	0.1557	− 0.56	0.573	− 0.3930	0.2175

q9_1	How often, if at all, would you use the following traditional sources to access COVID-19 updates?					Television	
		Coeff	SE	Z score	*p* value	95% Confidence interval	
Gender	Male	Reference					
	Female	0.0519	0.0832	0.62	0.533	− 0.1113	0.2150
Age group	18–39	Reference					
	40–59	− 0.6109	0.1064	− 5.74	0.000	− 0.8195	− 0.4022
	60 +	− 1.0029	0.1108	− 9.06	0.000	− 1.2200	− 0.7859
Ethnicity	White	Reference					
	BAME	− 0.1000	0.1262	− 0.79	0.428	− 0.3473	0.1473
Social group	ABC1	Reference					
	C2DE	− 0.0471	0.0915	− 0.51	0.607	− 0.2263	0.1322
Education	Low	− 0.3215	0.1229	− 2.62	0.009	− 0.5624	− 0.0806
	Medium	− 0.1522	0.0954	− 1.59	0.111	− 0.3392	0.0349
	High	Reference					
Region	South	Reference					
	North	− 0.3504	0.1086	− 3.23	0.001	− 0.5632	− 0.1376
	Midlands	− 0.3028	0.1067	− 2.84	0.005	− 0.5120	− 0.0936
	Scot, Wal, NI	0.0274	0.1311	0.21	0.834	− 0.2295	0.2843

q9_2	How often, if at all, would you use the following traditional sources to access COVID-19 updates?					Radio	
		Coeff	SE	Z score	*p* value	95% Confidence interval	
Gender	Male	Reference					
	Female	0.1449	0.0830	1.74	0.081	− 0.0179	0.3076
Age group	18–39	Reference					
	40–59	− 0.6250	0.1057	− 5.91	0.000	− 0.8322	− 0.4178
	60 +	− 0.5573	0.1102	− 5.06	0.000	− 0.7733	− 0.3413
Ethnicity	White	Reference					
	BAME	0.1600	0.1257	1.27	0.203	− 0.0863	0.4064
Social group	ABC1	Reference					
	C2DE	0.0891	0.0914	0.97	0.330	− 0.0901	0.2683
Education	Low	0.4706	0.1235	3.81	0.000	0.2286	0.7126
	Medium	0.0448	0.0954	0.47	0.638	− 0.1422	0.2319
	High	Reference					
Region	South	Reference					
	North	− 0.0876	0.1090	− 0.80	0.422	− 0.3014	0.1261
	Midlands	− 0.2649	0.1068	− 2.48	0.013	− 0.4743	-0.0555
	Scot, Wal, NI	− 0.0744	0.1292	− 0.58	0.565	− 0.3275	0.1788

q9_3	How often, if at all, would you use the following traditional sources to access COVID-19 updates?					Print tabloid newspapers/magazines	
		Coeff	SE	Z score	*p* value	95% Confidence interval	
Gender	Male	Reference					
	Female	0.1458	0.0970	1.50	0.133	− 0.0443	0.3359
Age group	18–39	Reference					
	40–59	− 0.2488	0.1304	− 1.91	0.056	− 0.5045	0.0068
	60 +	− 0.8712	0.1303	− 6.69	0.000	− 1.1265	− 0.6158
Ethnicity	White	Reference					
	BAME	− 0.6434	0.1458	− 4.41	0.000	− 0.9292	− 0.3576
Social group	ABC1	Reference					
	C2DE	− 0.0643	0.1057	− 0.61	0.543	− 0.2714	0.1428
Education	Low	− 0.8453	0.1411	− 5.99	0.000	− 1.1219	-0.5686
	Medium	− 0.5594	0.1165	− 4.80	0.000	− 0.7877	-0.3310
	High	Reference					
Region	South	Reference					
	North	− 0.1049	0.1274	− 0.82	0.411	− 0.3547	0.1449
	Midlands	− 0.1911	0.1228	− 1.56	0.120	− 0.4317	0.0495
	Scot, Wal, NI	0.0156	0.1564	0.10	0.921	− 0.2910	0.3222

q9_4	How often, if at all, would you use the following traditional sources to access COVID-19 updates?					Print broadsheet newspapers	
		Coeff	SE	Z score	*p* value	95% Confidence interval	
Gender	Male	Reference					
	Female	0.2679	0.0899	2.98	0.003	0.0918	0.4441
Age group	18–39	Reference					
	40–59	− 0.2993	0.1164	− 2.57	0.010	− 0.5274	− 0.0712
	60 +	− 0.7542	0.1199	− 6.29	0.000	− 0.9891	− 0.5193
Ethnicity	White	Reference					
	BAME	− 0.3560	0.1335	− 2.67	0.008	− 0.6177	− 0.0943
Social group	ABC1	Reference					
	C2DE	0.2966	0.1011	2.93	0.003	0.0985	0.4948
Education	Low	0.6394	0.1362	4.69	0.000	0.3724	0.9064
	Medium	0.3303	0.1029	3.21	0.001	0.1287	0.5320
	High	Reference					
Region	South	Reference					
	North	0.0704	0.1184	0.59	0.552	− 0.1616	0.3024
	Midlands	0.1005	0.1156	0.87	0.385	− 0.1261	0.3270
	Scot, Wal, NI	0.0446	0.1420	0.31	0.753	− 0.2336	0.3229

q10	Which, if either, of the following sources do you prefer to use to gather information on COVID-19?						
		Coeff	SE	Z score	*p* value	95% Confidence interval	
Gender	Male	Reference					
	Female	− 0.0327	0.1051	− 0.31	0.756	− 0.2386	0.1733
Age group	18–39	Reference					
	40–59	0.7266	0.1273	5.71	0.000	0.4770	0.9762
	60 +	1.6095	0.1407	11.44	0.000	1.3338	1.8853
Ethnicity	White	Reference					
	BAME	0.0685	0.1538	0.45	0.656	− 0.2329	0.3699
Social group	ABC1	Reference					
	C2DE	0.2629	0.1169	2.25	0.025	0.0338	0.4919
Education	Low	0.5237	0.1572	3.33	0.001	0.2156	0.8318
	Medium	0.2198	0.1177	1.87	0.062	− 0.0109	0.4506
	High	Reference					
Region	South	Reference					
	North	0.4455	0.1383	3.22	0.001	0.1744	0.7166
	Midlands	0.0746	0.1345	0.55	0.579	− 0.1890	0.3382
	Scot, Wal, NI	− 0.1791	0.1635	− 1.10	0.273	− 0.4996	0.1414

q11_1	To what extent, if at all, do you trust the COVID-19 information you receive from:					NHS Website	
		Coeff	SE	Z score	*p* value	95% Confidence interval	
Gender	Male	Reference					
	Female	− 0.0250	0.1029	− 0.24	0.808	− 0.2266	0.1766
Age group	18–39	Reference					
	40–59	0.1723	0.1290	1.34	0.182	− 0.0805	0.4251
	60 +	0.2669	0.1352	1.97	0.048	0.0020	0.5319
Ethnicity	White	Reference					
	BAME	0.2919	0.1478	1.98	0.048	0.0023	0.5815
Social group	ABC1	Reference					
	C2DE	0.4568	0.1101	4.15	0.000	0.2409	0.6727
Education	Low	0.3499	0.1480	2.36	0.018	0.0598	0.6400
	Medium	0.1572	0.1188	1.32	0.186	− 0.0757	0.3901
	High	Reference					
Region	South	Reference					
	North	− 0.0542	0.1334	− 0.41	0.685	− 0.3156	0.2073
	Midlands	0.0344	0.1302	0.26	0.792	− 0.2207	0.2895
	Scot, Wal, NI	− 0.2754	0.1670	− 1.65	0.099	− 0.6027	0.0519

q11_2	To what extent, if at all, do you trust the COVID-19 information you receive from:					Other, non-NHS, healthcare websites	
		Coeff	SE	Z score	*p* value	95% Confidence interval	
Gender	Male	Reference					
	Female	− 0.0565	0.0930	− 0.61	0.543	− 0.2388	0.1257
Age group	18–39	Reference					
	40–59	− 0.1654	0.1153	− 1.44	0.151	− 0.3914	0.0605
	60 +	0.3121	0.1218	2.56	0.010	0.0734	0.5507
Ethnicity	White	Reference					
	BAME	− 0.2725	0.1391	− 1.96	0.050	− 0.5451	0.0002
Social group	ABC1	Reference					
	C2DE	0.2236	0.1042	2.15	0.032	0.0194	0.4277
Education	Low	0.2266	0.1380	1.64	0.101	− 0.0440	0.4971
	Medium	0.0475	0.1054	0.45	0.652	− 0.1592	0.2541
	High	Reference					
Region	South	Reference					
	North	0.1049	0.1203	0.87	0.383	− 0.1309	0.3406
	Midlands	− 0.2370	0.1203	− 1.97	0.049	− 0.4729	-0.0011
	Scot, Wal, NI	− 0.0277	0.1470	− 0.19	0.850	− 0.3159	0.2604

q11_3	To what extent, if at all, do you trust the COVID-19 information you receive from:					Tabloid news websites	
		Coeff	SE	Z score	*p* value	95% Confidence interval	
Gender	Male	Reference					
	Female	− 0.0557	0.0941	− 0.59	0.554	− 0.2401	0.1287
Age group	18–39	Reference					
	40–59	− 0.0147	0.1183	− 0.12	0.901	− 0.2465	0.2172
	60 +	− 0.5060	0.1234	− 4.10	0.000	− 0.7479	− 0.2641
Ethnicity	White	Reference					
	BAME	− 0.3464	0.1392	− 2.49	0.013	− 0.6193	− 0.0735
Social group	ABC1	Reference					
	C2DE	− 0.2544	0.1037	− 2.45	0.014	− 0.4577	− 0.0512
Education	Low	− 0.6805	0.1388	− 4.90	0.000	− 0.9525	− 0.4085
	Medium	− 0.6597	0.1080	− 6.11	0.000	− 0.8713	− 0.4480
	High	Reference					
Region	South	Reference					
	North	0.0197	0.1225	0.16	0.872	− 0.2203	0.2597
	Midlands	− 0.0512	0.1199	− 0.43	0.670	− 0.2862	0.1838
	Scot, Wal, NI	0.2600	0.1512	1.72	0.085	− 0.0362	0.5563

q11_4	To what extent, if at all, do you trust the COVID-19 information you receive from:					Broadsheet news websites	
		Coeff	SE	Z score	*p* value	95% Confidence interval	
Gender	Male	Reference					
	Female	0.0475	0.0928	0.51	0.609	− 0.1344	0.2293
Age group	18–39	Reference					
	40–59	− 0.0116	0.1140	− 0.10	0.919	− 0.2350	0.2119
	60 +	− 0.0329	0.1201	− 0.27	0.784	− 0.2683	0.2025
Ethnicity	White	Reference					
	BAME	− 0.2663	0.1376	− 1.94	0.053	− 0.5360	0.0034
Social group	ABC1	Reference					
	C2DE	0.3693	0.1025	3.60	0.000	0.1684	0.5702
Education	Low	1.0575	0.1409	7.50	0.000	0.7812	1.3337
	Medium	0.6312	0.1049	6.02	0.000	0.4255	0.8369
	High	Reference					
Region	South	Reference					
	North	0.3204	0.1217	2.63	0.008	0.0818	0.5590
	Midlands	0.2463	0.1182	2.08	0.037	0.0146	0.4780
	Scot, Wal, NI	0.2141	0.1441	1.49	0.137	− 0.0684	0.4966

q11_5	To what extent, if at all, do you trust the COVID-19 information you receive from:					BBC news website	
		Coeff	SE	Z score	*p* value	95% Confidence interval	
Gender	Male	Reference					
	Female	0.0152	0.0888	0.17	0.864	− 0.1589	0.1892
Age group	18–39	Reference					
	40–59	0.0859	0.1099	0.78	0.434	− 0.1295	0.3014
	60 +	− 0.0140	0.1150	− 0.12	0.903	− 0.2394	0.2115
Ethnicity	White	Reference					
	BAME	− 0.2507	0.1317	− 1.90	0.057	− 0.5089	0.0075
Social group	ABC1	Reference					
	C2DE	0.4022	0.0985	4.08	0.000	0.2090	0.5953
Education	Low	0.4702	0.1319	3.56	0.000	0.2116	0.7287
	Medium	0.3655	0.1006	3.63	0.000	0.1683	0.5628
	High	Reference					
Region	South	Reference					
	North	− 0.1055	0.1165	− 0.91	0.365	− 0.3340	0.1229
	Midlands	− 0.0035	0.1128	− 0.03	0.975	− 0.2246	0.2176
	Scot, Wal, NI	− 0.0110	0.1380	− 0.08	0.937	− 0.2814	0.2594

q11_6	To what extent, if at all, do you trust the COVID-19 information you receive from:					Social media	
		Coeff	SE	Z score	*p* value	95% Confidence interval	
Gender	Male	Reference					
	Female	− 0.3378	0.0927	− 3.64	0.000	− 0.5195	− 0.1561
Age group	18–39	Reference					
	40–59	0.0686	0.1123	0.61	0.541	− 0.1515	0.2887
	60 +	0.4424	0.1205	3.67	0.000	0.2062	0.6786
Ethnicity	White	Reference					
	BAME	− 0.6974	0.1357	− 5.14	0.000	− 0.9634	− 0.4314
Social group	ABC1	Reference					
	C2DE	0.0461	0.1020	0.45	0.651	− 0.1538	0.2460
Education	Low	− 0.6170	0.1393	− 4.43	0.000	− 0.8900	− 0.3440
	Medium	− 0.2970	0.1042	− 2.85	0.004	− 0.5011	− 0.0928
	High	Reference					
Region	South	Reference					
	North	0.2275	0.1199	1.90	0.058	− 0.0075	0.4625
	Midlands	0.1044	0.1203	0.87	0.385	− 0.1313	0.3402
	Scot, Wal, NI	− 0.1350	0.1395	− 0.97	0.333	− 0.4084	0.1385

q12	How confident are you in telling apart reliable COVID-19 information online or through apps?						
		Coeff	SE	Z score	*p* value	95% Confidence interval	
Gender	Male	Reference					
	Female	0.1048	0.0962	1.09	0.276	− 0.0838	0.2934
Age group	18–39	Reference					
	40–59	− 0.0945	0.1168	− 0.81	0.419	− 0.3233	0.1344
	60 +	0.2637	0.1263	2.09	0.037	0.0161	0.5113
Ethnicity	White	Reference					
	BAME	0.1883	0.1378	1.37	0.172	− 0.0818	0.4584
Social group	ABC1	Reference					
	C2DE	0.3390	0.1058	3.21	0.001	0.1317	0.5463
Education	Low	0.4029	0.1425	2.83	0.005	0.1237	0.6821
	Medium	0.1202	0.1083	1.11	0.267	− 0.0920	0.3325
	High	Reference					
Region	South	Reference					
	North	− 0.0299	0.1247	− 0.24	0.810	− 0.2743	0.2145
	Midlands	− 0.1126	0.1233	− 0.91	0.361	− 0.3543	0.1290
	Scot, Wal, NI	− 0.2432	0.1505	− 1.62	0.106	− 0.5382	0.0517

q13_1	If you saw information on COVID-19, which of the following would contribute towards your trust in it?					That it comes from the Government	
		Coeff	Std. Err	Z score	*p* value	95% Confidence interval	
Gender	Male	Reference					
	Female	0.0044	0.0934	0.05	0.962	− 0.1786	0.1875
Age group	18–39	Reference					
	40–59	− 0.2408	0.1182	− 2.04	0.042	− 0.4725	− 0.0091
	60 +	− 0.1385	0.1213	− 1.14	0.253	− 0.3763	0.0992
Ethnicity	White	Reference					
	BAME	− 0.0633	0.1391	− 0.46	0.649	− 0.3359	0.2093
Social group	ABC1	Reference					
	C2DE	− 0.1099	0.1030	− 1.07	0.286	− 0.3118	0.0919
Education	Low	0.1641	0.1372	1.20	0.232	− 0.1049	0.4330
	Medium	0.0774	0.1075	0.72	0.472	− 0.1334	0.2882
	High	Reference					
Region	South	Reference					
	North	− 0.0307	0.1223	− 0.25	0.802	− 0.2704	0.2090
	Midlands	0.0175	0.1188	0.15	0.883	− 0.2154	0.2505
	Scot, Wal, NI	− 0.1789	0.1474	− 1.21	0.225	− 0.4678	0.1101

q13_2	If you saw information on COVID-19, which of the following would contribute towards your trust in it?					That it comes from scientists/scientific institutions	
		Coeff	SE	Z score	*p* value	95% Confidence interval	
Gender	Male	Reference					
	Female	0.1400	0.1056	1.33	0.185	− 0.0670	0.3470
Age group	18–39	Reference					
	40–59	− 0.1364	0.1368	− 1.00	0.319	− 0.4044	0.1317
	60 +	− 0.1413	0.1405	− 1.01	0.314	− 0.4167	0.1340
Ethnicity	White	Reference					
	BAME	− 0.5234	0.1559	− 3.36	0.001	− 0.8290	− 0.2178
Social group	ABC1	Reference					
	C2DE	− 0.3815	0.1116	− 3.42	0.001	− 0.6002	− 0.1628
Education	Low	− 1.0539	0.1535	− 6.87	0.000	− 1.3547	− 0.7530
	Medium	− 0.6077	0.1295	− 4.69	0.000	− 0.8615	− 0.3540
	High	Reference					
Region	South	Reference					
	North	− 0.2404	0.1403	− 1.71	0.087	− 0.5153	0.0346
	Midlands	− 0.3917	0.1342	− 2.92	0.004	− 0.6548	− 0.1286
	Scot, Wal, NI	− 0.1932	0.1655	− 1.17	0.243	− 0.5177	0.1312

q13_6	If you saw information on COVID-19, which of the following would contribute towards your trust in it?					The source it comes from	
		Coeff	SE	Z score	*p* value	95% Confidence interval	
Gender	Male	Reference					
	Female	− 0.1464	0.0990	− 1.48	0.139	− 0.3404	0.0476
Age group	18–39	Reference					
	40–59	− 0.4019	0.1209	− 3.33	0.001	− 0.6388	− 0.1650
	60 +	− 0.7401	0.1291	− 5.73	0.000	− 0.9930	− 0.4871
Ethnicity	White	Reference					
	BAME	− 0.0372	0.1423	− 0.26	0.794	− 0.3162	0.2417
Social group	ABC1	Reference					
	C2DE	− 0.2930	0.1112	− 2.64	0.008	− 0.5109	− 0.0752
Education	Low	− 0.8875	0.1501	− 5.91	0.000	− 1.1817	− 0.5933
	Medium	− 0.6311	0.1099	− 5.74	0.000	− 0.8464	− 0.4157
	High	Reference					
Region	South	Reference					
	North	− 0.2000	0.1300	− 1.54	0.124	− 0.4549	0.0548
	Midlands	− 0.2475	0.1271	− 1.95	0.052	− 0.4966	0.0017
	Scot, Wal, NI	− 0.0121	0.1534	− 0.08	0.937	− 0.3127	0.2885

q14	How often do you double check online or app-based health information that you receive?						
		Coeff	SE	Z score	*p* value	95% Confidence interval	
Gender	Male	Reference					
	Female	− 0.1068	0.0964	− 1.11	0.268	− 0.2958	0.0822
Age group	18–39	Reference					
	40–59	− 0.1628	0.1159	− 1.41	0.160	− 0.3899	0.0642
	60 +	0.1477	0.1289	1.15	0.252	− 0.1050	0.4004
Ethnicity	White	Reference					
	BAME	− 0.1274	0.1343	− 0.95	0.343	− 0.3907	0.1358
Social group	ABC1	Reference					
	C2DE	0.3778	0.1097	3.44	0.001	0.1627	0.5928
Education	Low	0.8407	0.1511	5.56	0.000	0.5445	1.1369
	Medium	0.1489	0.1076	1.38	0.166	− 0.0619	0.3597
	High	Reference					
Region	South	Reference					
	North	0.0520	0.1258	0.41	0.679	− 0.1945	0.2986
	Midlands	0.0600	0.1242	0.48	0.629	− 0.1834	0.3035
	Scot, Wal, NI	0.0104	0.1517	0.07	0.945	− 0.2870	0.3078

q15	How likely are you to engage with digital resources if they were directly linked to the controlling the pandemic?						
		Coeff	SE	Z score	*p* value	95% Confidence interval	
Gender	Male	Reference					
	Female	− 0.1072	0.0821	− 1.31	0.192	− 0.2681	0.0537
Age group	18–39	Reference					
	40–59	0.0973	0.1012	0.96	0.336	− 0.1010	0.2956
	60 +	0.3608	0.1071	3.37	0.001	0.1509	0.5707
Ethnicity	White	Reference					
	BAME	0.2321	0.1198	1.94	0.053	− 0.0027	0.4669
Social group	ABC1	Reference					
	C2DE	0.4160	0.0903	4.61	0.000	0.2390	0.5930
Education	Low	0.1579	0.1199	1.32	0.188	− 0.0771	0.3928
	Medium	0.0178	0.0937	0.19	0.849	− 0.1658	0.2014
	High	Reference					
Region	South	Reference					
	North	0.2887	0.1082	2.67	0.008	0.0768	0.5007
	Midlands	0.0410	0.1037	0.40	0.693	− 0.1622	0.2442
	Scot, Wal, NI	0.2440	0.1275	1.91	0.056	− 0.0059	0.4939

q16_1	How comfortable are you in sharing the following personal data with a Government COVID-19 contact tracing app?					NHS number	
		Coeff	SE	Z score	*p* value	95% Confidence interval	
Gender	Male	Reference					
	Female	0.2449	0.0822	2.98	0.003	0.0838	0.4061
Age group	18–39	Reference					
	40–59	0.1674	0.1031	1.62	0.104	− 0.0346	0.3695
	60 +	− 0.1668	0.1063	− 1.57	0.116	− 0.3751	0.0414
Ethnicity	White	Reference					
	BAME	0.1770	0.1211	1.46	0.144	− 0.0604	0.4145
Social group	ABC1	Reference					
	C2DE	0.1285	0.0895	1.44	0.151	− 0.0469	0.3040
Education	Low	0.1292	0.1196	1.08	0.280	− 0.1052	0.3637
	Medium	− 0.0076	0.0943	− 0.08	0.935	− 0.1924	0.1771
	High	Reference					
Region	South	Reference					
	North	0.0569	0.1075	0.53	0.597	− 0.1538	0.2677
	Midlands	− 0.0437	0.1037	− 0.42	0.673	− 0.2469	0.1595
	Scot, Wal, NI	0.0877	0.1294	0.68	0.498	− 0.1659	0.3413

q16_2	How comfortable are you in sharing the following personal data with a Government COVID-19 contact tracing app?					Age	
		Coeff	SE	Z score	*p* value	95% Confidence interval	
Gender	Male	Reference					
	Female	0.1095	0.0844	1.30	0.195	− 0.0560	0.2750
Age group	18–39	Reference					
	40–59	0.1224	0.1054	1.16	0.245	− 0.0841	0.3289
	60 +	− 0.1541	0.1100	− 1.40	0.161	− 0.3696	0.0615
Ethnicity	White	Reference					
	BAME	0.2155	0.1220	1.77	0.077	− 0.0236	0.4546
Social group	ABC1	Reference					
	C2DE	0.1332	0.0922	1.44	0.149	− 0.0476	0.3139
Education	Low	0.0580	0.1234	0.47	0.638	− 0.1838	0.2997
	Medium	− 0.0732	0.0969	− 0.76	0.450	− 0.2631	0.1167
	High	Reference					
Region	South	Reference					
	North	0.0548	0.1104	0.50	0.620	− 0.1616	0.2711
	Midlands	0.0899	0.1066	0.84	0.399	− 0.1191	0.2989
	Scot, Wal, NI	0.0658	0.1329	0.50	0.621	− 0.1946	0.3262

q16_3	How comfortable are you in sharing the following personal data with a Government COVID-19 contact tracing app?					Location	
		Coeff	SE	Z score	*p* value	95% Confidence interval	
Gender	Male	Reference					
	Female	0.1284	0.0827	1.55	0.120	− 0.0336	0.2904
Age Group	18–39	Reference					
	40–59	− 0.2813	0.1037	− 2.71	0.007	− 0.4845	− 0.0781
	60 +	− 0.6367	0.1079	− 5.90	0.000	− 0.8481	− 0.4252
Ethnicity	White	Reference					
	BAME	0.2632	0.1204	2.19	0.029	0.0272	0.4992
Social Group	ABC1	Reference					
	C2DE	0.2122	0.0901	2.36	0.018	0.0357	0.3888
Education	Low	0.0716	0.1205	0.59	0.553	− 0.1646	0.3077
	Medium	0.0403	0.0950	0.42	0.671	− 0.1459	0.2264
	High	Reference					
Region	South	Reference					
	North	− 0.0373	0.1083	− 0.34	0.731	− 0.2495	0.1750
	Midlands	− 0.0062	0.1041	− 0.06	0.952	− 0.2103	0.1979
	Scot, Wal, NI	0.1129	0.1289	0.88	0.381	− 0.1397	0.3654

q16_4	How comfortable are you in sharing the following personal data with a Government COVID-19 contact tracing app?					Medical history	
		Coeff	SE	Z score	*p* value	95% Confidence interval	
Gender	Male	Reference					
	Female	0.0879	0.0819	1.07	0.283	− 0.0726	0.2484
Age group	18–39	Reference					
	40–59	0.1331	0.1031	1.29	0.197	− 0.0689	0.3352
	60 +	− 0.1849	0.1059	− 1.75	0.081	− 0.3925	0.0226
Ethnicity	White	Reference					
	BAME	0.0222	0.1206	0.18	0.854	− 0.2141	0.2586
Social group	ABC1	Reference					
	C2DE	0.0707	0.0900	0.79	0.432	− 0.1057	0.2471
Education	Low	− 0.1372	0.1210	− 1.13	0.257	− 0.3744	0.1000
	Medium	− 0.1391	0.0939	− 1.48	0.138	− 0.3230	0.0449
	High	Reference					
Region	South	Reference					
	North	0.0393	0.1074	0.37	0.715	− 0.1713	0.2498
	Midlands	− 0.0922	0.1033	− 0.89	0.372	− 0.2947	0.1103
	Scot, Wal, NI	0.0864	0.1285	0.67	0.502	− 0.1656	0.3383

q17_1	How comfortable are you in sharing the following personal data with an industry-led COVID-19 contact tracing app?					NHS number	
		Coeff	SE	Z score	*p* value	95% Confidence interval	
Gender	Male	Reference					
	Female	0.1600	0.0865	1.85	0.064	− 0.0095	0.3296
Age group	18–39	Reference					
	40–59	0.0164	0.1087	0.15	0.880	− 0.1966	0.2294
	60 +	0.1777	0.1128	1.58	0.115	− 0.0434	0.3989
Ethnicity	White	Reference					
	BAME	− 0.1019	0.1296	− 0.79	0.432	− 0.3560	0.1521
Social group	ABC1	Reference					
	C2DE	− 0.0358	0.0951	− 0.38	0.707	− 0.2222	0.1507
Education	Low	0.0090	0.1275	0.07	0.944	− 0.2410	0.2589
	Medium	− 0.0089	0.0995	− 0.09	0.929	− 0.2038	0.1860
	High	Reference					
Region	South	Reference					
	North	0.1277	0.1147	1.11	0.266	− 0.0971	0.3526
	Midlands	− 0.1239	0.1087	− 1.14	0.254	− 0.3370	0.0891
	Scot, Wal, NI	0.1038	0.1364	0.76	0.447	− 0.1634	0.3710

q17_2	How comfortable are you in sharing the following personal data with an industry-led COVID-19 contact tracing app?					Age	
		Coeff	SE	Z score	*p* value	95% Confidence interval	
Gender	Male	Reference					
	Female	0.1224	0.0825	1.48	0.138	− 0.0393	0.2840
Age group	18–39	Reference					
	40–59	0.2048	0.1035	1.98	0.048	0.0019	0.4077
	60 +	0.6356	0.1070	5.94	0.000	0.4258	0.8453
Ethnicity	White	Reference					
	BAME	0.1611	0.1244	1.29	0.195	− 0.0827	0.4048
Social group	ABC1	Reference					
	C2DE	− 0.0308	0.0897	− 0.34	0.732	− 0.2066	0.1451
Education	Low	0.1245	0.1205	1.03	0.301	− 0.1116	0.3606
	Medium	0.1097	0.0940	1.17	0.243	− 0.0746	0.2940
	High	Reference					
Region	South	Reference					
	North	0.2006	0.1080	1.86	0.063	− 0.0110	0.4122
	Midlands	0.0565	0.1038	0.54	0.586	− 0.1470	0.2599
	Scot, Wal, NI	− 0.0382	0.1299	− 0.29	0.769	− 0.2928	0.2165

q17_3	How comfortable are you in sharing the following personal data with an industry-led COVID-19 contact tracing app?					Location	
		Coeff	SE	Z score	*p* value	95% Confidence interval	
Gender	Male	Reference					
	Female	0.2100	0.0828	2.54	0.011	0.0477	0.3723
Age group	18–39	Reference					
	40–59	− 0.0181	0.1039	− 0.17	0.861	− 0.2217	0.1854
	60 +	0.3386	0.1075	3.15	0.002	0.1280	0.5492
Ethnicity	White	Reference					
	BAME	0.2095	0.1244	1.68	0.092	− 0.0343	0.4534
Social group	ABC1	Reference					
	C2DE	0.0834	0.0900	0.93	0.354	− 0.0930	0.2597
Education	Low	0.1403	0.1213	1.16	0.248	− 0.0975	0.3782
	Medium	0.0161	0.0949	0.17	0.865	− 0.1699	0.2021
	High	Reference					
Region	South	Reference					
	North	0.1173	0.1092	1.07	0.283	− 0.0967	0.3313
	Midlands	− 0.0254	0.1048	− 0.24	0.809	− 0.2309	0.1801
	Scot, Wal, NI	− 0.0473	0.1294	− 0.37	0.715	− 0.3008	0.2063

q17_4	How comfortable are you in sharing the following personal data with an industry-led COVID-19 contact tracing app?					Medical history	
		Coeff	SE	Z score	*p* value	95% Confidence interval	
Gender	Male	Reference					
	Female	− 0.0029	0.0872	− 0.03	0.973	− 0.1738	0.1680
Age group	18–39	Reference					
	40–59	0.0872	0.1096	0.80	0.426	− 0.1276	0.3020
	60 +	0.2972	0.1136	2.62	0.009	0.0746	0.5198
Ethnicity	White	Reference					
	BAME	− 0.1052	0.1310	− 0.80	0.422	− 0.3619	0.1515
Social group	ABC1	Reference					
	C2DE	− 0.0718	0.0954	− 0.75	0.452	− 0.2587	0.1152
Education	Low	− 0.1227	0.1291	− 0.95	0.342	− 0.3757	0.1304
	Medium	− 0.1177	0.1003	− 1.17	0.240	− 0.3142	0.0788
	High	Reference					
Region	South	Reference					
	North	0.0615	0.1153	0.53	0.594	− 0.1645	0.2874
	Midlands	− 0.1427	0.1095	− 1.30	0.192	− 0.3573	0.0719
	Scot, Wal, NI	0.0362	0.1375	0.26	0.792	− 0.2332	0.3056

### Access

99% (2024/2040) of the sample cohort have access to a personal digital device (Question 1). Smartphones and laptops/personal computers have the highest penetrance at 88% (1788/2040) and 84% (1719/2040) across the cohort respectively. 61% (1239/2040) of the cohort own tablet computers. Smartwatches (211/2040, 10%) and wearable fitness trackers (391/2040, 19%) were less frequently owned by respondents.

With respect to age, access to personal computers/laptops is stable through to the 60+ age group (651/746 (87%) in 18–39 age group compared to 522/615 (85%) in the 60+ age group). In contrast, smartphone ownership declines in the 60+ age group (702/746 (94%) in the 18–39 age group compared to 465/615 (76%) in the 60 + age group). Ownership of laptops/personal computers decline with lower social grade (508/571 (89%) in AB compared to 337/449 (75%) in DE). Smartphone ownership declines with lower educational attainment groups (587/634 (93%) in the high educational attainment group compared to 434/535 (81%) in the low educational attainment group).

836/2024 (41%) of respondents state that they have used their personal digital device to access COVID-19 specific information (Question 1.1). This figure decreases with age (372/740 (50%) between ages 18 and 39 compared to 182/609 (30%) in those aged above 60), social grades (274/568 (48%) in AB compared to 145/442 (33%) in DE) and educational attainments cohorts (329/632 (52%) in the high educational attainment group compared to 160/529 (30%) in the low educational attainment group). Of all personal digital device activities, instant messaging (1652/2024 (82%)) was the most commonly utilised function, followed by accessing the news (1476/2024 (73%)), telephone calls (1461/2024 (72%)) and then social networking (1447/2024 (71%)).

### Confidence

1423/2040 (70%) are confident at using online or app-based information to make personal health decisions (Question 2). In comparison to their reference counterparts, respondents who are female, over the age of 60 and of a lower social grade are all significantly less confident in using online or app-based information to make personal health decisions (*p* < 0.01) (Question 2). Those above the age of 60 are consistently significantly less confident in both sourcing and using health resources to form personal health decisions regardless of digital source (internet, apps or social media (Questions 5, 6 and 7) (*p* < 0.01) and would rather consult a clinician over the phone than an online or app-based telemedicine service (*p* < 0.01) (Question 3). Those from lower social grades and of lower educational attainment are significantly less confident at knowing where (Question 6.1) and how (Question 5.1) to use the internet to answer health questions (*p* < 0.01). There are no significant consistent findings with respect to either ethnicity or region for this domain of questions.

**Figure 2:**
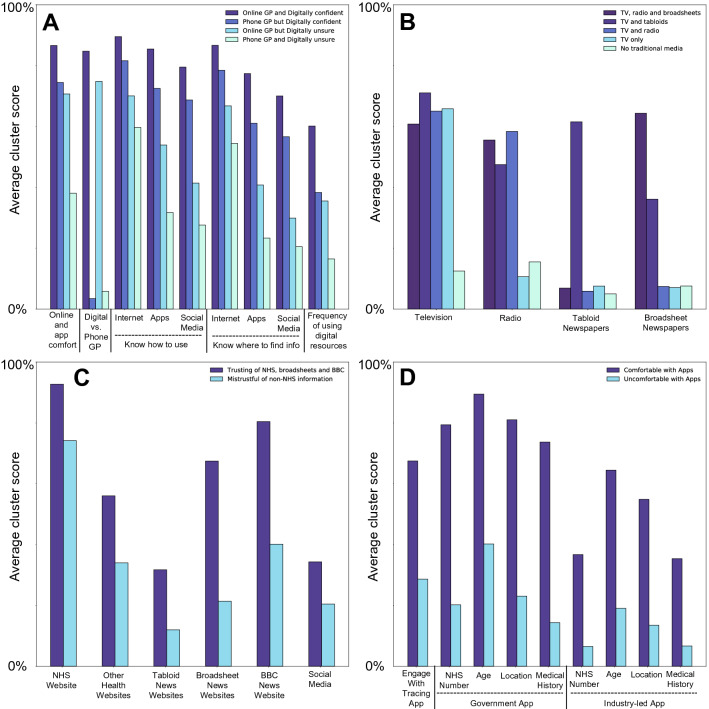
4 bar graphs (labelled Panel A, B, C and D) detailing discrete response types within survey domains, achieved through K-means cluster scores.

Four distinct clusters of responses for this domain of questions (Questions 3, 5 and 6) were identified. Panel A of Fig. 2 shows the responses of each cluster to each of the constituent questions on which clustering is performed. Clusters were characterised post-hoc based on their responses as ‘Digitally confident and preferring online primary care’ (19%), ‘Digitally confident and preferring telephone primary care’ (34%), ‘Digitally cautious and preferring online primary care’ (24%) and ‘Digitally cautious and preferring telephone primary care’ (23%).

### Sources of information

Respondents over the age of 40, from lower social grades and of lower educational attainment use online or app-based resources less often than their reference counterparts (*p* < 0.01) (Question 7). 675/2040 (34%) have not used online resources or apps to seek any COVID-19 information at all (Question 7). Over three times as many people over the age of 60 (124/259 (42%) compared to 95/746 (13%)) in the 18–39 age group would rather access health information from traditional (non-digital) media sources than relying upon digital media sources (Question 10). Those above the age of 60 are more likely to turn towards tabloid newspapers, broadsheet newspapers radio and television than their references counterparts (*p* < 0.01) whilst avoiding social media (*p* < 0.01). Those of lower social grades and educational attainment are less likely to use broadsheet newspaper sources (paper or online format) (*p* < 0.01) (Questions 8 and 9). Respondents of BAME background are also more likely to engage in many digital (non-NHS websites, tabloid newspaper website, broadsheet website, social media) and traditional information sources (print tabloid and broadsheet newspapers) (p < 0.01) than reference counterparts (Questions 8 and 9).

Five distinct clusters of responses for this domain of questions (Question 9) were identified. Panel B of Fig. 2 shows the responses of each cluster to each of the constituent questions on which clustering is performed. Clusters were characterised post-hoc based on their source of information preference; ‘TV, radio and broadsheets’ (12.3%), ‘TV and radio’ (25.7%), TV and tabloids’ (14.8%), ‘TV only’ (26.4%) and ‘No traditional media’ (20.7%).

### Trust

885/2040 (43%) cited ‘trust in the information found’ as the main barrier against the use of online/app-based information to guide personal health decisions, ahead of ‘knowing where to find information’ (406/2040 (20%)) and ‘knowing how to action the information found’ (379/2040 (19%)) (Question 4). Those above the age of 60 (*p* < 0.05), from lower social grades (*p* < 0.01) and of lower educational attainment (*p* < 0.01) are less confident in telling apart reliable COVID-19 information from unreliable information when encountered online or through apps (Question 12).

Amongst information sources, the NHS website has the highest trust rating (1661/2040 (81%)) whereas social media (1325/2040 (65%)) and tabloid newspapers (1303/2040 (64%)) has the highest distrust rating (Question 11). However, the NHS website is not as preferred by those in lower social grades (*p* < 0.01), those of low educational attainment (*p* < 0.05), those above 60 (*p* < 0.05) and those of BAME backgrounds (*p* < 0.05). In addition, broadsheet newspaper sources and the BBC are not as trusted as information sources by those from low social grades and low educational attainment groups (*p* < 0.01).

Two distinct clusters of responses for this domain of questions (Question 11) were identified. Panel C of Fig. 2 shows the responses of each cluster to each of the constituent questions on which clustering is performed. Clusters were characterised post-hoc based on their responses as either ‘mistrustful of non-NHS information’ (37.5%) or ‘Trusting of NHS, broadsheets and BBC’ (62.5%).

Scientific endorsement of information from figures, such as Professor Chris Whitty, is seen as the most important contributor towards trust (70% trust rating). Despite this high rating, in comparison to their reference groups, respondents from BAME backgrounds, lower social grades, low educational attainment groups and those who reside in the Midlands are less likely to trust information that has scientific endorsement. Moreover, the government trust rating was only 40%, with no one demographic either more or less inclined to trust government sourced information in comparison to the reference group. Lastly, those with a high education attainment (213/634) are twice as likely to double check information that they encounter through digital resources than those of a low education attainment (80/535) (Question 14).

### Contact tracing

832/2040 (41%) are unlikely to engage with a digital contact tracing programme, even in the event that compliance was directly linked to easing of quarantine measures. In comparison to their respective reference groups, those above the age of 60 (*p* < 0.01), those from Northern regions (*p* < 0.01) and those of the lowest social grade are significantly less likely to engage in the contact tracing programme (*p* < 0.05) (Question 15).

With respect to industry led contact tracing apps, respondents are uncomfortable with sharing their NHS number (1524/2040 (75%)), medical history (1538/2040 (75%)) and location (1199/2040 (59%)). Those aged above 60 are significantly more uncomfortable in sharing data related to age, location and medical history when using industry led apps, in comparison to their reference counterparts (*p* < 0.01) (Question 17). In comparison, with respect to government led contact tracing apps, there is less discomfort at sharing NHS number (795/2040 (39%)), medical history (935/2040 (46%)) and location (772/3040 (38%)) (Question 16). With government led contact tracing apps, those of a BAME background and lower social grades are less comfortable in sharing their location than their reference counterparts (*p* < 0.05), whereas those over the 40+ are more likely to share their location (*p* < 0.01).

Two distinct clusters of responses for this domain of questions (Questions 15, 16 and 17) were identified. Panel D of Fig. 2 shows the responses of each cluster to each of the constituent questions on which clustering is performed. Clusters were characterised post-hoc based on their responses as either ‘comfortable with apps’ (59.3%) or ‘uncomfortable with apps’ (40.7%).

A Brant test was performed to test the proportional odds assumption with respect to each of the ordinal logistic regression models (Appendix 2). We note that the proportional odds assumption was valid except in Questions 2 and 12–17. No single covariate was consistently responsible for violation of the proportional odds assumption across these models. This is likely secondary to the large sample size as well as the high number of explanatory variables included in the models^25^.

## Discussion

This study finds that the UK population exhibits (1) diverse preferences for accessing public health information, (2) mixed self-rated ability to use digital health resources and (3) variable levels of engagement with digital public health approaches, resulting in incomplete digital inclusivity during the COVID-19 pandemic. This study has shown there is a consistent pattern of older people, those of lower social grades and those of lower educational attainment levels displaying greater vulnerability to digital exclusion through poorer access to devices, diminished ability to navigate digital resources pertaining to public health efforts, and reduced inclination to interact with them. In contrast, reported attitudes and behaviours amongst BAME groups are more complex, and do not uniformly align with risk for digital exclusion. With respect to the barriers to digital inclusion, the findings somewhat corroborate the high levels of internet and device availability in the UK as previously described^9^. However, our results also reveal disparities with respect the ability to use and engagement with digital solutions. These findings are particularly marked with regards to digital public health messaging, disease surveillance and contact tracing.

As this was an online survey, we did not expressly ask about internet connectivity, which would have been requisite for respondents. Early 2020 national data^8^ shows that 96% of the UK have internet access and whilst the remaining 4% have not been represented in this work, given they have no access, they would also not be able to engage with digital public health strategies, being the most digitally excluded. Our findings are, therefore, likely to be conservative estimate of the extent of digital exclusion amongst the UK population. Laptop, personal computer or phone access were relatively high across participants of all demographic groups and more frequently used than other device types. Whilst the pandemic has interrupted the publication of the full range of annual ONS data on this topic, these figures appear consistent with other sources^26^.

National data shows that internet connection in households with an adult aged over 65 years has increased to 80% this year and was predominantly used by the elderly for maintaining social interaction and online shopping prior to the pandemic^8^. Although our data show a continued trend in older, low social grade and lower educational attainment subpopulations using the internet for social interaction, this did not translate to many of these participants accessing digital COVID-19-related public health messaging or contact tracing apps. This discrepancy may be explained by the combination of lower self-reported ability to find and use such information, as well as concerns that participants raised about the reliability of online health information. Although these groups prefer television or print media for COVID-19 updates, and have a degree of mistrust of online resources, including government endorsed media, they continue to use digital devices for social media. Yet, familiarity with, and frequent use of, such platforms in combination with knowledge gaps in identifying reliable information leave people open to the spread of health misinformation^27^. Notable COVID-19-specific examples of misinformation have led to the destruction of 5G network towers^28^, case reports of ingested disinfectant^29^ and poor compliance with face masks^30^.

The study also reveals factors contributing to scant use of apps for COVID-19 disease surveillance or contact tracing. In the first instance, the elderly, those of lower social grades and of lower educational attainment had less smartphone access^31^, however, sentiments of trust and privacy played a greater role. Amongst the total study population, 41% report being unlikely to engage with such an app, citing reduced trust and concerns sharing health data with non-NHS private partners, such as Apple and Google. These trends were more pronounced still amongst older and those of lower social grades. This is interesting in view of the less secure centralised data storage option preferred by the UK government versus the decentralized but more secure alternative used by the tech giants^32^. This counterfactual highlights potential knowledge gaps but also the role of privacy and trust in encouraging digital inclusion^33^. Furthermore, these barriers to engagement undermine the efficacy of a contact tracing app which requires up to an estimated 60% uptake^34^, particularly in the absence of an operational test and trace system, as was the case in the UK at the time of the study being conducted^35^.

The picture of digital exclusion gleaned from this study is far more mixed for the BAME cohort. This is perhaps as BAME is an umbrella that encompasses much heterogeneity in cultural background, income level and education, all of which could have a greater effect on digital inclusion. As such, studying the attitudes and views of BAME people as a single group is unlikely to be an adequate approach^36^ and focus should be placed on engaging with those without English as a first language, who are recognised as being at risk from the digital divide^9^.

Although this is a UK-based study, the digital divide is by no means a UK-specific phenomenon. The United Nations Sustainable Development Goal 9.c of providing “universal and affordable access to the Internet in least developed countries by 2020” has not been met^37^. Despite modestly improving internet access rates globally, low digital literacy skills remain a barrier to meaningful participation in a digital society. It is therefore unsurprising that similarly themed studies conducted in countries as varied as Ghana^38^ and the Netherlands^39^ suggest that groups vulnerable to digital exclusion have struggled to locate and engage with COVID-19 information disseminated via digital media. This divide is also seen in public-facing clinical digital health interventions during the pandemic, namely tele-medicine services^40,41^.

Despite increasingly high levels of internet connection and device availability and the pandemic accelerating digital technology adoption, we report a gradient among older, lower social grades and lower education attainment demographic groups interacting with digital public health approaches. The inability to promptly access and understand online information and services prevents individuals from taking protective steps against COVID-19. These same groups are also at higher risk from COVID-19, so the observed digital divide effectively compounds health risks. This suggests that digital inequality potentiates vulnerability to the pandemic, thereby further increasing health inequalities. This is in keeping with previous descriptions of digital inclusion as a wider determinant of health^42,43^.

## Recommendations

Failing to consider how digital interventions can exacerbate health inequalities could be disastrous. Instead, previous national commitments to alleviate digital exclusion^44^ should be reaffirmed. The clustering of responses reveals a lack of consensus across key issues of acquisition and consumption of digital healthcare data, implying that there is unlikely to be a ‘one-size-fits-all’ digital strategy to provide equitable coverage across all regions and populations. As such, a multifaceted response, targeting the barriers to digital inclusion is essential.

### Access

Though we found relatively high levels of connectivity within our cohort, attention should be given to emerging groups who struggle with slow connection speeds or expensive internet service provision that impede education or employment. We did not study children’s experiences but governmental programmes to provide either new or refurbished^45^ laptops and internet connection to children^46^ provides multigenerational support to engage in digital health services^47^.

### Skills

Closer collaboration between the technology sector, non-governmental organisations and governmental stakeholders can produce solutions that are scalable and robust. For example, in the USA, Microsoft have provided funding and infrastructural support to provide both devices and access to digital skills training to the Public Library Association^48^. Integration of digital skills assessments within routine services, such as GP services, can also help identify individuals who are at risk of the digital divide and would require support.

### Engagement

Greater direct communication between digital service providers and communities can assuage mistrust. The NHS Widening Digital Participation Programme^49^ trains ‘digital champions’ who are trusted community members and able to provide support to less confident members of the community group^50^. Similarly contact-tracing app developers can and have increased trust and uptake through public information campaigns to improve understanding and transparency in lay terms^51^.

Whilst many of these strategies are primarily framed at bridging the digital divide during the COVID-19 pandemic, there is evidence to suggest that laying the groundwork for greater digital inclusion will pay dividends in the post-COVID-19 era in improving health and social equality. However, whilst these strategies are being introduced, it is essential that non-digital options, such as telephone services and staffed public access points, must remain available for those who are unable to engage with digital services.

### Limitations

The sampling methodology employed by YouGov is both a strength and limitation of the study. The non-probabilistic method employed allowed for the prompt and cost-effective delivery of a prespecified sample size from segments of the population, who are traditionally difficult to engage in qualitative research. This method, however, precludes nonresponse bias calculations, and harbours a higher degree of bias than probabilistic sampling. Additionally, this cross-sectional survey provides a snapshot of people’s preferences, rather than how sentiments evolve over time. Public trust in entities, such as government, varies over the course of a crisis, and could provide some explanation for the low government net trust rating (40%)^52^. The study data did not include comorbidities of respondents therefore exploration of this group, who are potentially vulnerable to COVID-19, could not be performed. Furthermore, the YouGov survey is also unlikely to have accessed proportionate numbers of marginalised people such as migrant workers, the homeless and sex-workers who are at risk of COVID-19, and have poor access to healthcare and digital interventions^42,53,54^. In addition, as noted, those without internet access will also not have been able to participate in the study.

## Conclusion

This study demonstrates an ongoing digital divide in the UK population with older, groups of lower social grade and educational attainment reporting less preparedness for COVID-19 digital health strategies. It highlights how a ‘digital first’ model of disseminating critical health information, disease surveillance and digital contact tracing have significant potential to marginalise population groups who are concurrently vulnerable to both digital exclusion and poor health outcomes secondary to SARS-CoV-2.

Given the importance of maintaining low transmission rates across all regions and population groups, there is an urgent need for key decision makers to consider further investment in multifaceted strategies to mitigate this possibility. Solutions should be targeted towards the principal drivers of digital exclusion; (1) access, (2) skills and (3) engagement. Through the empowerment of end-users, public health strategies will have a greater chance of containing disease spread and limiting the deepening of inequalities in health outcomes and the digital divide.

## Supplementary Information

Supplementary Information 1.

Supplementary Information 2.
