# Supplementary material for: A national survey assessing public readiness for digital health strategies against COVID-19 within the United Kingdom
Source: Sci Rep. 2021 Mar 16;11:5958. doi: 10.1038/s41598-021-85514-w (PMC7966397; doi:10.1038/s41598-021-85514-w)
Supplement: Supplementary file 1 — Supplementary Information 1. [file 41598_2021_85514_MOESM1_ESM.docx]

**A National Survey Assessing Public Readiness for Digital Health Strategies Against COVID-19 within the United Kingdom**

**Authors:**

Viknesh Sounderajah* ^1,2^, Jonathan Clarke* ^1,2,3^, Seema Yalamanchili ^1,2^, Amish Acharya ^1,2^, Sheraz R. Markar ^1^, Hutan Ashrafian ^1,2^, Ara Darzi ^1,2^

*joint first author

**Affiliations:**

1. Department of Surgery and Cancer, Imperial College London, London, W2 1NY, United Kingdom
2. Institute of Global Health Innovation, Imperial College London, London, W2 1NY, United Kingdom
3. Department of Mathematics, Imperial College London, London, SW7 2AZ, United Kingdom

YouGov Questions

| Number | Question | E-Health Literacy Framework Domain | COVID-19 Specific Domain |
| --- | --- | --- | --- |
| 1  1.1 | BASE QUESTION: Which, if any, of the following personal digital devices do you have access to?   1. Smartphone 2. Smartwatch 3. Wearable fitness tracker (e.g. FitBit) 4. Tablet 5. Laptop/PC 6. Other 7. Not applicable – I do not have access to any personal digital devices.   BRANCH QUESTION: What have you used your personal digital device(s) for during the COVID-19 pandemic?   1. Video calling 2. Telephone calls 3. Texting/instant messaging 4. Social networking 5. Work-related activities 6. Accessing the news 7. Accessing COVID-19 specific advice 8. Other, please specify 9. Don’t know | Access (6) | Demographics |
| 2 | How comfortable are you in using online or app-based information to make personal health decisions?   1. Very comfortable 2. Somewhat comfortable 3. Somewhat uncomfortable 4. Very uncomfortable 5. Don’t know | Engagement in health (2) | Confidence |
| 3 | Assuming you are unable to talk to a doctor face to face, would you prefer to use an online/app-based tool (e.g. video calls or consultations with a doctor through a private health service app), or would you rather have consult a doctor over the phone?   1. I would prefer to use an online/app-based tool 2. I would prefer to have a consultation over the phone 3. No preference 4. Don’t know | Motivation (5) | Confidence |
| 4 | Which barriers, if any, affect your use of online or app-based services to guide your health decisions?   1. Access to a personal digital device (e.g. smartphone, tablet or computer) 2. Perception that it takes too long 3. Trust in the information found 4. Knowing where to find information 5. Knowing how to action the information found 6. Don’t know 7. Other, please specify 8. Not applicable – there are no barriers to my use of online or app-based services | Ability to actively engage with digital services (3) |  |
| 5 | To what extent do you agree or disagree with the following statements? (strongly disagree to strongly agree)   1. I know how to use the internet to answer questions about my health 2. I know how to use apps to answer questions about my health 3. I know how to use social media to answer questions about my health | Ability to actively engage with digital services (3)  Ability to process information (1) | Confidence |
| 6 | To what extent do you agree or disagree with the following statements? (strongly disagree to strongly agree)   1. I know where helpful health resources are available on the internet 2. I know where helpful health resources are available on apps 3. I know where helpful health resources are available on social media | Services to suit need (7)  Ability to engage (3) | Confidence |
| 7 | When you have accessed COVID-19 specific health information, how often would you use online or app-based health resources? (If you have not accessed COVID-19 information on the internet, please select the “Not applicable” option). (Never to All the time) | Ability to engage (3) | Confidence |
| 8 | How often, if at all, would you use the following digital sources to access COVID-19 updates? (Never to All the time)   1. NHS Website 2. Other, non-NHS, healthcare websites 3. Tabloid news websites (e.g. The Daily Mail, The Daily Express, The Sun, etc.) 4. Broadsheet news websites (e.g. The Telegraph, The Guardian, etc.) 5. BBC news website 6. Social media (e.g. Facebook, Twitter, etc.) |  | Source |
| 9 | How often, if at all, would you use the following traditional (non-digital) sources to access COVID-19 updates? (Never to all the time)   1. Television 2. Radio 3. Print tabloid newspapers/magazines (e.g. The Daily Mail, The Daily Express, The Sun etc.) 4. Print broadsheet newspapers (e.g. The Telegraph, The Guardian, etc.) |  | Source |
| 10 | Which, if either, of the following sources do you prefer to use to gather information on COVID-19? (If you have not been seeking COVID-19 information, please select the “Not applicable” option).   1. Digital media (e.g. websites, social networking platforms, apps) 2. Traditional (non-digital) media (e.g. newspapers, radio, television) 3. No preference 4. Don’t know 5. Not applicable |  | Source |
| 11 | To what extent, if at all, do you trust the COVID-19 information you receive from the following digital sources? (Not at all to a lot)   1. NHS Website 2. Other, non-NHS, healthcare websites 3. Tabloid news websites (e.g. The Daily Mail, The Daily Express, etc.) 4. Broadsheet news websites (e.g. The Telegraph, The Guardian, etc.) 5. BBC news website 6. Social media (e.g. Facebook, Twitter, etc.) |  | Trust |
| 12 | How confident are you in telling apart reliable COVID-19 information from unreliable COVID-19 information online or through apps?   1. Very confident 2. Somewhat confident 3. Somewhat unconfident 4. Very unconfident 5. Don’t know 6. Not applicable – I would not look for COVID-19 information on the internet |  |  |
| 13 | If you saw information on COVID-19, which, if any, of the following things would contribute towards your trust in it? (Please select all that apply. If nothing would contribute towards your trust, please select the “Not applicable” option).   1. That it comes from the Government 2. That it comes from scientists/scientific institutions (e.g. Professor Chris Witty) 3. That it comes from celebrities 4. That it comes from friends or family members 5. The source it comes from (e.g. newspaper, website, television) 6. Other, please specify 7. Don’t know 8. Not applicable – no factors increase my trust in COVID-19 information | Safety and control (4) |  |
| 14 | How often do you double check online or app-based health information that you receive? (Never to always) | Safety and control (4) |  |
| 15 | How likely are you to engage with digital health resources (e.g. downloading contact tracing app on smartphones) if they were directly linked to the controlling the pandemic? (very likely to very unlikely) | Ability to actively engage with digital services (3) |  |
| 16 | How comfortable are you in sharing the following personal data with Government led COVID-19 contact tracing apps? (very comfortable to very uncomfortable)   1. NHS number 2. Age 3. Location 4. Medical history | Safety and control (4) | Contact Tracing |
| 17 | How comfortable are you in sharing the following personal data with industry led (e.g. Google/Apple) COVID-19 contact tracing apps? (very comfortable to very uncomfortable)   1. NHS number 2. Age 3. Location 4. Medical history | Safety and control (4) | Contact Tracing |
